# What incentives encourage local communities to collect and upload mosquito sound data by using smartphones? A mixed methods study in Tanzania

**DOI:** 10.1186/s41256-023-00298-y

**Published:** 2023-05-29

**Authors:** Rinita Dam, Winifrida Mponzi, Dickson Msaky, Tumpe Mwandyala, Emmanuel W. Kaindoa, Marianne E. Sinka, Ivan Kiskin, Eva Herreros-Moya, Janey Messina, Syed Ghulam Sarwar Shah, Stephen Roberts, Kathy J. Willis

**Affiliations:** 1 Department of Biology, University of Oxford, Oxford, UK; 2 Warwick Medical School, University of Warwick, Warwick, UK; 3 Environmental Health and Ecological Science Department, Ifakara Health Institute, P.O. Box 53, Ifakara, Tanzania; 4 The Nelson Mandela, African Institution of Science and Technology, School of Life Sciences and Bioengineering, Tengeru, Arusha Tanzania; 5 Department of Engineering Science, University of Oxford, Oxford, UK; 6 Surrey Institute for People-Centred AI, Centre for Vision Speech and Signal Processing, University of Surrey, Guildford, UK; 7 School of Geography and the Environment and the Oxford School of Global and Area Studies, University of Oxford, Oxford, UK; 8 NIHR Oxford Biomedical Research Centre, Oxford University Hospitals NHS Foundation Trust, Oxford, UK

**Keywords:** Mosquito surveillance, Acoustic recognition, HumBug sensor, Digital citizen science, Community engagement, Mixed methods study

## Abstract

**Background:**

To detect and identify mosquitoes using their characteristic high-pitched sound, we have developed a smartphone application, known as the ‘HumBug sensor’, that records the acoustic signature of this sound, along with the time and location. This data is then sent remotely to a server where algorithms identify the species according to their distinctive acoustic signature. Whilst this system works well, a key question that remains is what mechanisms will lead to effective uptake and use of this mosquito survey tool? We addressed this question by working with local communities in rural Tanzania and providing three alternative incentives: money only, short message service (SMS) reminders and money, and SMS reminders only. We also had a control group with no incentive.

**Methods:**

A multi-site, quantitative empirical study was conducted in four villages in Tanzania from April to August 2021. Consenting participants (n = 148) were recruited and placed into one of the three intervention arms: monetary incentives only; SMS reminders with monetary incentives; and SMS reminders only. There was also a control group (no intervention). To test effectiveness of the mechanisms, the number of audio uploads to the server of the four trial groups on their specific dates were compared. Qualitative focus group discussions and feedback surveys were also conducted to explore participants’ perspectives on their participation in the study and to capture their experiences of using the HumBug sensor.

**Results:**

Qualitative data analysis revealed that for many participants (37 out of 81), the main motivation expressed was to learn more about the types of mosquitoes present in their houses. Results from the quantitative empirical study indicate that the participants in the ‘control’ group switched on their HumBug sensors more over the 14-week period (8 out of 14 weeks) when compared to those belonging to the ‘SMS reminders and monetary incentives’ trial group. These findings are statistically significant (p < 0.05 *or* p > 0.95 under a two-sided z-test), revealing that the provision of monetary incentives and sending SMS reminders did not appear to encourage greater number of audio uploads when compared to the control.

**Conclusions:**

Knowledge on the presence of harmful mosquitoes was the strongest motive for local communities to collect and upload mosquito sound data via the HumBug sensor in rural Tanzania. This finding suggests that most efforts should be made to improve flow of real-time information back to the communities on types and risks associated with mosquitoes present in their houses.

**Supplementary Information:**

The online version contains supplementary material available at 10.1186/s41256-023-00298-y.

## Background

Mosquito-borne diseases are major contributors to the global burden of infectious disease, as they include both very high burden and important emerging diseases, such as human malaria (around 212 million cases per year), dengue (around 96 million cases per year), chikungunya (around 693,000 cases per year), and Zika virus disease (around 500, 000 cases per year) [1, 2]. The ever-increasing availability of innovative sensor technology and sophisticated software, however, have made it possible to create better, safer and cheaper mosquito surveillance and control systems to help mitigate this problem [1]. For example, citizen science projects have engaged members of the public to record data, such as specific types of mosquito occurrence, via applications on their mobile phones [3, 4]. The HumBug sensor was specifically designed to identify malaria vectors, with the idea that this system could enhance malaria vector surveillance, specifically in locations that are hard to access. Current surveillance methods are time consuming, expensive and can put the surveyor’s life at risk (e.g., human landing catches) [5]. As a result, this technology has the potential to allow significantly greater levels of mosquito surveillance over both time and space. Using a HumBug sensor could allow longitudinal surveillance to identify where interventions are failing, highlight real time changes in mosquito abundance and, with the correct training data for the detection and identification algorithms, identify invading species (e.g., *An. stephensi*).

Mobile phones are ubiquitous and are relatively accessible in sub-Saharan Africa (SSA) [6]. Data published by Groupe Spéciale Mobile Association (GSMA) in 2018, for example, revealed that 44% of the population of SSA are mobile phone subscribers, and this number is predicted to go up to 50% by 2025 [7]. Mobile internet penetration in SSA has also achieved substantial growth over a period of only four years, from 13% in 2014 to 24% in 2018, which is predicted to increase up to 39% by 2025 [8] with at least 7% of the population in SSA with 4G connections [8]. Smartphone connection in SSA is predicted to increase up to 55% by 2025 [9].

The proliferation of mobile phones and good 4G coverage have prompted a range of innovative applications using phones for mobile personal health care, commonly known as mHealth [10]. The modern smartphone is ideal for delivering mHealth because it contains a suite of programmable sensors (e.g. camera, light sensor, proximity sensor, microphone, digital compass, accelerometer and Global Positioning System) that can also be used to collect behavioural and physiological data [10]. A number of applications (apps) for health and well-being have consequently emerged in the past few years where built-in mobile phone sensors generate health data [11]. These apps are essentially transforming smartphones into medical devices to capture data and deliver advice that can be used by health professionals [12, 13], by patients and the general public [13]. For example, within the context of the UK, in 2001, the National Health Service (NHS) devised the concept of the ‘Expert Patient’ after evaluating numerous small-scale self-management programmes, mostly relating to chronic diseases such as diabetes, which revealed that participants were able to cope with their illnesses when they were encouraged to self-manage their conditions, thus becoming ‘experts’ on their own condition [14]. In the context of malaria specifically, mobile phones have been used in a variety of ways. For example, SMS based reporting has been used to monitor stocks of life-saving and antimalarial medicines, monitor presence of suboptimal drugs and rapid diagnostic test kits, as well as, detecting mosquito-related disease outbreaks [15–23]. Apps have also been developed to record data on the presence of mosquitoes. One study, for example, used a mobile phone-based system (called Chaak) to monitor the early stage (larvae) of dengue mosquitoes in artificial containers on individual premises. The number of immature stages of *Aedes aegypti* L. present per type of container (e.g. buckets, tyres, cisterns) and each house were uploaded via surveyors’ mobile phones to the central repository of the Chaak system using Wi-Fi connection [24].

In all these applications, however, a key question to emerge is how to ensure that people use their smartphones as health and data collection devices? SMS reminders are used more commonly but, in some cases, small monetary incentives have also been trialled. In a study in Malawi, for example, small monetary incentives were provided to people on the condition that they modify behaviour or complete the desired activity of HIV testing [25]. Results from a cluster randomised controlled trial in rural Kenya also revealed that the deployment of SMS reminders *plus* monetary incentives significantly improved childhood immunisation coverage compared to the control group [26]. A study in rural Kenya and rural Tanzania revealed that SMS based reporting coupled with small monetary payments was better at incentivising health facility workers to provide timely and correct stock count of antimalarial drugs in comparison to just SMS messages [15, 20].

Other studies, however, have indicated that people may participate in these type of citizen science projects because of intrinsic motivations [27]. Intrinsic motivations describe the desire for the volunteer to participate because they find volunteering inherently interesting or satisfying. Intrinsic motivations to participate in citizen science projects include a desire to learn new things and share existing knowledge with others [28], to help science and other people [29] and to help the environment [30]. In a recent malaria study in rural Rwanda [31], for example results demonstrated that there were four motivational factors for participating: curiosity; a desire to learn new things; helping other people; and contributing to malaria control [31]. Exploration into the motivational factors for participation in citizen science projects has mainly been conducted in North America, Europe and Australia; to date there is very little empirical evidence available in the context of Asia, Africa and Central America [32].

Over the past five years, we have developed a novel acoustic mosquito survey tool called HumBug, which uses a budget smartphone running our MozzWear app to detect mosquitoes using their characteristic buzz. The current system passively records host-seeking mosquitoes as they are attracted to people sleeping under an adapted bednet (HumBug Net) and subsequently, requires the homeowner to participate in the deployment of the smartphone in a bednet at night time, and then upload the data to a server the following day [33].

The objective of this study therefore was to determine which incentive mechanism and/or intrinsic motivational factor (s) would lead to the most effective uptake and use of the HumBug survey tool by local communities in developing countries.

## Methods

### Study setting and design

**Fig. 1:**
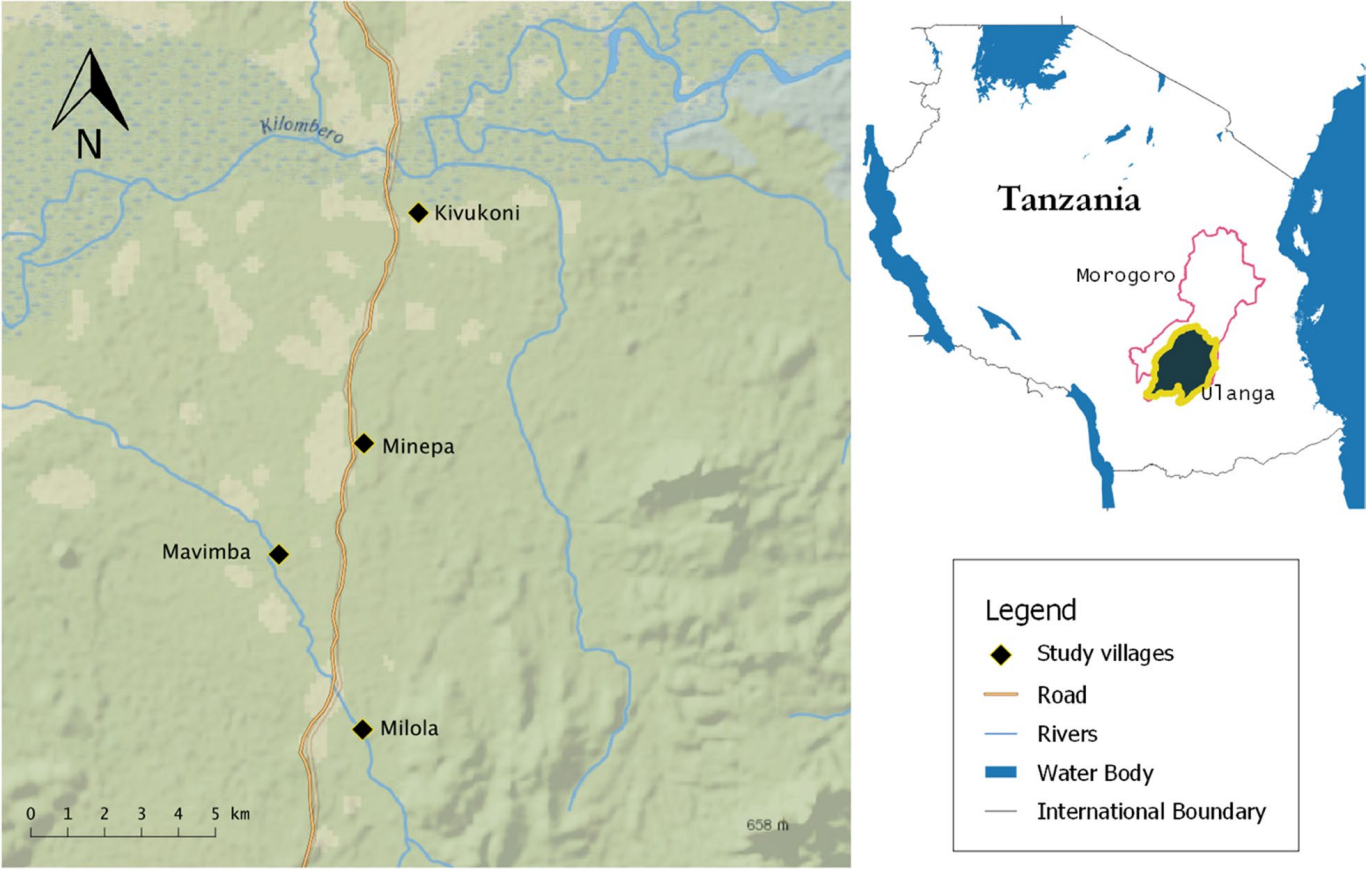
Map showing the location of the four study sites

The study was conducted in four rural villages (Kivukoni, Minepa, Mavimba and Milola) located within the Ulanga district in south-east Tanzania (Fig. 1). The four villages lie 270 m above sea level with an annual rainfall of 1200–1800 mm and a temperature range of 20–38 °C. This area has a high mosquito density. The dominant malaria vectors are *Anopheles funestus sensu stricto* (s.s.) and *Anopheles arabiensis* [34–37]. The main economic activity in this area is subsistence rice cultivation using irrigation systems [38].

We conducted a multi-site, quantitative empirical study combined with pre-trial participant focus group discussions (FGDs) and post-trial feedback surveys. In all four study areas, heads of households (or a member of household who was willing to participate in the study) were placed into one of three trial groups: (i) provision of monetary incentives only (via airtime scratch cards for mobile phones); (ii) monetary incentives plus SMS reminders; (iii) SMS reminders only. We also had a no-incentive control group.

The quantitative empirical study ran for 14 weeks (from 23 April 2021 to 15 August 2021). Mosquito flight tone data uploads began from 24 April 2021 to 16 August 2021. The study included three stages. In stage one, qualitative data were collected via focus group discussions to explore people’s perceptions of participating in the study; in stage two, empirical data were collected to investigate whether the application of monetary incentives (monetary incentives and SMS reminders or SMS reminders only), encouraged people to switch on their HumBug sensors, record, and upload mosquito sound data. Finally, to receive feedback from all the participants who took part in stages one and two, the third stage involved a feedback survey administered to capture the participants’ experiences of using the HumBug sensor. Following the methods of Fetters et al. [39], data collected from the three stages were analysed separately but combined in the reporting.

### Data collection and tools

#### Stage one: qualitative study

During the first phase of the study, focus group participants were drawn from Kivukoni, Minepa, Mavimba and Milola villages within the Ulanga district in Tanzania (Fig. 1). The participants were selected to cover a range of age, gender, education level and profession. The focus group discussions (FGDs) were conducted between November 2020 and February 2021. Purposive sampling strategies were used to recruit 37 participants from each of the four villages, with a total of 148 participants.

Community members were eligible to participate if they were residents of the selected four villages, aged 18 or over, own or have access to a personal mobile phone (where it is possible to transfer monetary incentive and/or receive SMS reminders), and were willing to provide a signed informed consent prior to the study. The village leaders were guided to recruit a diverse sample of community members with respect to demographics (including gender, education level and family size). Maximum variation sampling and snowball sampling techniques were used to identify participants with relevant experiences and ensure a sufficiently diverse sample [40].

Community members from each village were invited to participate in focus group discussions (FGDs). Focus groups took place in either empty classrooms in primary schools or community centres in the four villages and typically lasted 80 min. A community engagement researcher (WM) led the facilitation of the focus groups in Kiswahili and a research assistant (TM) and, an experienced qualitative health researcher (RD), acted as co-facilitators. All participants were asked to complete a short questionnaire to capture demographic information about themselves and their family. Topic guides were used to guide discussions, exploring their understanding of how the HumBug sensor was going to be set up in the HumBug Net in their homes [33]; their understanding of how they were going to be allocated to a trial group; and what were the factors that would motivate them to take part in the study (see Additional file 1 and Additional file 2: Focus group/interview guide to engage community members in rural Tanzania in English and a translated version in Kiswahili, respectively). Data collection continued until saturation was reached, with interviewees providing no substantively new information. The focus group discussions that were conducted in November 2020, were audio (digitally) recorded with the consent of participants, transcribed verbatim and translated from Kiswahili to English.

#### Stage two: quantitative empirical study

**Fig. 2:**
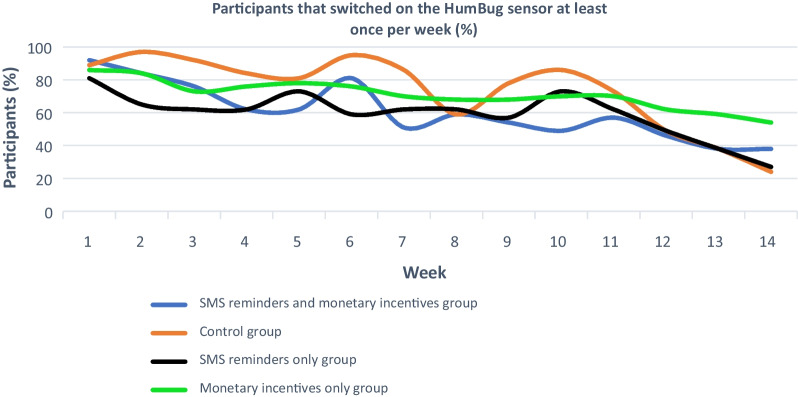
Results from the percentage of participants that switched on their HumBug sensors and uploaded mosquito sound data on specific days

**Fig. 3:**
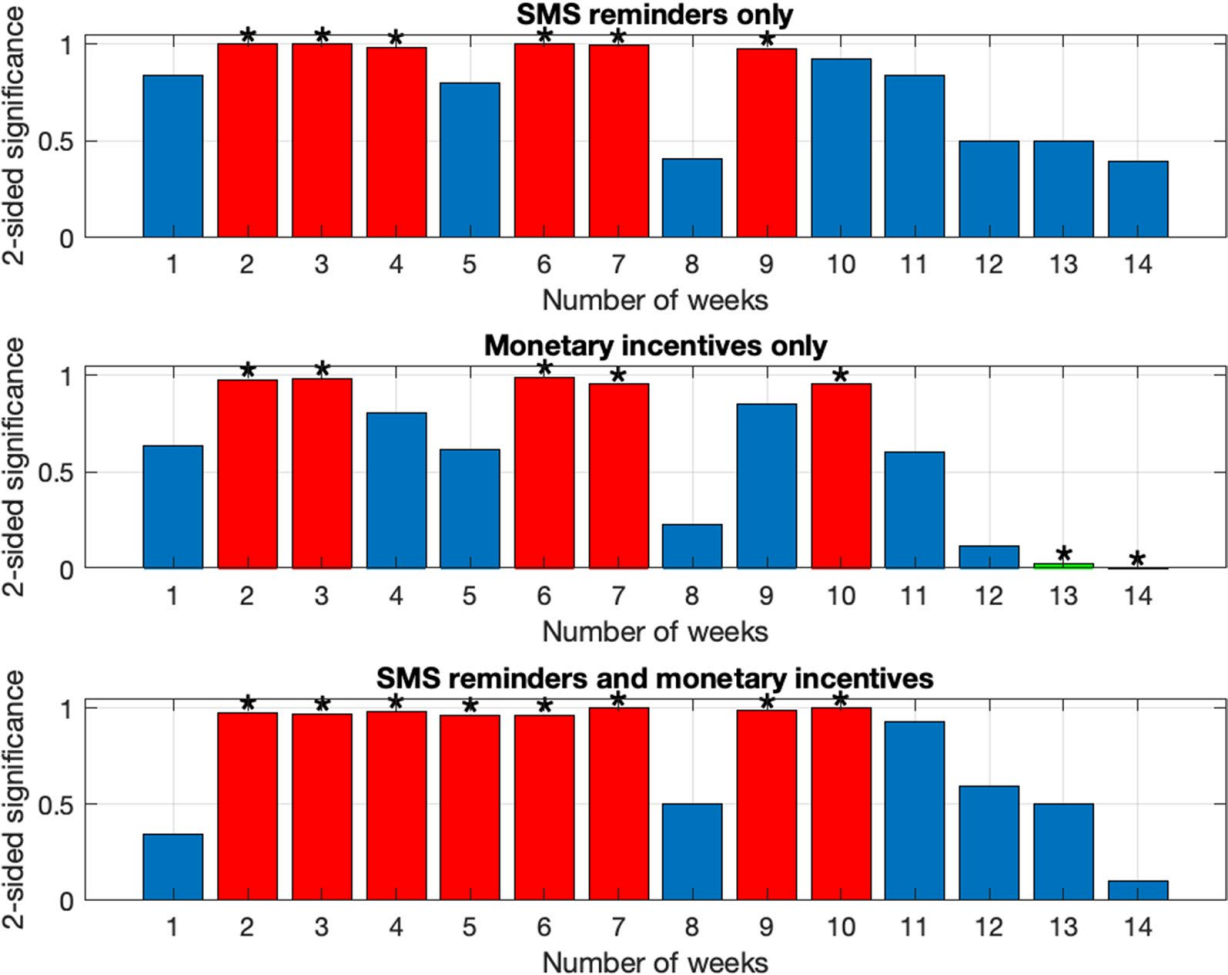
Comparison of the three intervention groups against the control group. Results from our two-sided z-test scores comparing each of the three intervention groups against the control group indicate that those in the control group switched on their HumBug sensors more than those in the three intervention groups. The p-values indicate if a particular week in either of the three intervention groups had significantly more ‘1s’ (i.e., they switched on their HumBug sensors that week) than the control group. The red bars in the figure indicate p > 0.95, implying that the participants in the control group had significantly *more* ‘1s’ than those in the three intervention groups. The green bars (present only at weeks 13 and 14 in the ‘Monetary incentives only’ chart), represent p < 0.05, meaning that the participants in the intervention group had significantly *more* ‘1s’ than those in the control group. The * markers are placed on top of the bars encoded in red and green to indicate that these findings are statistically significant (p < 0.05 *or* p > 0.95 under a two-sided z-test). The blue bars represent data being within the two-sided z-test critical values [0.05, 0.95], enabling observation of any trends, but the values are not statistically significant

The second phase of the study was to conduct an evaluation and compare the provision of ‘monetary incentives only’, ‘SMS reminders only’ and ‘SMS reminders and monetary incentives’ with a control situation (without any incentive), in the four village communities in Tanzania over 14 weeks (April to August 2021), we investigated the following:

1. The number of people in the four trial groups switching on their Humbug sensors at least once on the requested date throughout the trial period (Fig. 2),
2. Ascertain statistical significance of activity between participants belonging to the control group, compared to those belonging to the three intervention groups (Fig. 3).

To achieve our objective, the 148 participants (37 participants from each village) described above were provided with a study information sheet in Kiswahili. Written consent was obtained from the community members who wished to take part in the trial.

The four villages were randomly assigned into four trial groups: Kivukoni (SMS reminders and monetary incentives); Minepa (control); Mavimba (SMS reminders only) and Milola (monetary incentives only). To select which village was assigned to each trial group, a public ceremony took place in Minepa, where 10 representatives and village leaders from all the four villages were present. As part of the ceremony, the names of the trial groups were written on pieces of paper, placed in a bag and shuffled. A representative from each village then drew a piece of paper at random from the bag and after, was allocated to the selected trial group.

Once each community was assigned to a trial group, all 148 participants were provided with a budget smartphone running the Mozzwear application (HumBug sensor), a training course on how to use the HumBug sensor,along with a new SIM card for the sensor, and a new mosquito net (HumBug Net) with a pocket where the smartphone was placed overnight, designed to allow the flight tones of host-seeking mosquitoes to be passively captured. The HumBug sensor required the data to be manually uploaded (synced) to the server.

The quantitative empirical study (stage two) began on 23 April 2021. SMS reminders were sent out to the 74 people (belonging to the ‘SMS reminders only’ and ‘SMS reminders and monetary incentives’ trial groups) on a weekly basis. At the end of each month, monetary incentives in the form of airtime scratch cards for mobile phones with a value of $10 (equivalent to 23,000 Tanzanian Shillings) were provided to the 74 participants (belonging to the ‘monetary incentives only’ and ‘SMS reminders and monetary incentives’ groups). Once a week, on specific dates, all of 148 participants belonging to the four trial groups, were asked to place the smartphone in their HumBug Net, switch it on and allow it to record overnight (from 6 pm to 6 am), and then sync the data in the morning. Once a month, the local research team in Tanzania visited the participants in all the trial groups to provide them with $2 (equivalent to 4,600 Tanzanian Shillings) worth of money so that the participants could buy data which they needed to continue uploading mosquito sound data at least once a week throughout the 14-week trial period. The study came to an end on 15 August 2021 and the final set of mosquito sound data uploads were obtained on 16 August 2021. The mosquito sound data recordings that are received and stored in a MongoDB database, were accessed via a dashboard that showed device recording identification number as well as visual representation of the audio [33].

Shortly after the quantitative empirical study (stage two) began in the four villages, a questionnaire was conducted with all 148 participants to capture key demographics. The variables used in this questionnaire were adapted from the household questionnaire found on the Demographic and Health Survey (DHS) Program website [41] (for the adapted version of the questionnaire used in this study, see Additional file 3 and Additional file 4: Demographic Questionnaire in English and a translated version in Kiswahili). Demographic questionnaire data were collected in electronic form using Open Data Kit (ODK) which is an open-source mobile data collection software for resource-limited settings [42, 43]. Demographic questionnaire data were collected from all 148 participants via door-to-door visits.

#### Stage three: quantitative feedback survey

The final phase of the study involved conducting a feedback survey with all 148 participants that took part in the quantitative empirical study (stage two). (Please see Table 1 for the demographics of the sample). Written consent was obtained from these participants before the data were collected. Similar to the demographic questionnaire, data for the feedback were collected using ODK and the survey was conducted between 22 and 30 July 2021 to capture participants’ experiences of using the HumBug sensor and of taking part in the trial. Participants were asked to express how much they agreed or disagreed with a particular question related to the study, and this was done on a six-point Likert scale: “Strongly agree” (score 5), “Agree” (score 4), “Not sure” (score 3), “Disagree” (score 2), “Strongly disagree” (score 1), and “Question not asked” (score 0). Participants were also asked to provide some demographic characteristics (e.g., gender, age, and profession). In addition to the quantitative questions, the survey contained two open-ended questions that asked participants to share their views on (1) “what was positive about their experience?” and (2) “what would have made their research experience better?”.

The questionnaire was developed by reviewing previously published research [44, 45], and adapted to the local context (See Additional file 5 and Additional file 6 for ‘Research Participant Experience Survey’ in English and a translated version in Kiswahili, respectively).

### Data analysis

#### Stage one: qualitative study

To analyse the responses from the FGDs, eight transcripts were imported into NVivo software (version 12) and analysed thematically [46]. RD and WM, both trained and experienced in conducting qualitative research analysis, independently conducted qualitative coding, where they systematically categorised segments in the transcripts, in order to find themes and patterns. RD and WM read the transcripts, identified emergent themes, created initial codes, brought codes together to create a coding framework and coded the transcripts with NVivo 12. RD and WM conducted constant comparison, an iterative method of analysis, searching for each themed code throughout the entire data set and comparing all instances until no new themes were identified. Emerging findings were discussed via video conferences to resolve discrepancies and refine themes.

#### Stage two: quantitative empirical study

To find out how many people in the four trial groups switched on their HumBug sensors at least once over the 14-week period and uploaded mosquito sound data, we performed a simple count of the number of participants who switched on their sensors on each day and uploaded data as recorded by the participants’ sensor identification numbers using the dashboard.

To determine whether there was any statistically significant difference in the smartphone activity of participants belonging to the control group compared to those belonging to the three intervention groups, we performed statistical analysis using bespoke software written in a combination of the Python and Octave programming languages [47, 48]. In this analysis, data uploads were represented as binary [1,0], corresponding to a switching on the phone, recording and uploading mosquito sound data having taken place, or not. Primary statistical analysis then considered the data as sets of draws from Binomial distributions. Given the control data set (sites with no incentives), we considered the significance of deviations in the intervention groups from the statistics of the control set by evaluating the differences between two Binomial sample sets. By noting that, even for modest sample sizes, normal approximations to the Binomial may be used, we were then able to exploit Boschloo’s test for Binomials as an Exact z-Pooled Test [49] simplifying our significance testing to that of critical-value analysis in a standard z-test.

#### Stage three: quantitative feedback survey

Participants’ response data were downloaded from ODK on to a Microsoft Excel spreadsheet format. Data were summarised using frequencies and descriptive analysis was conducted on the responses and shown as percentages. Table 3.1 to 3.5 present the percentage of participants providing feedback at the end of the trial.

## Results

### Stage one: qualitative study

The participants were selected to cover a range of age, gender, education level and profession. Participants ranged in age (from 18 to 69 years); they included both women and men; their education levels ranged from primary to tertiary schooling, and they had varied professions including farming, owning their own business, and working as a tailor.

A summary of participants recruited is provided in Table 1:

**Table 1:** Demographics of the sample

Community members	n (range)
*Gender*	
Female	74
Male	74
*Age*	
Mean (range) years	37.9 (18–69)
*Education status*	
Primary	102
Secondary	34
Tertiary	12
*No. of children*	
Mean (range)	2.1 (0–9)
*Profession*	
Farmer only	102
Business owner only	19
Teacher	9
Farmer and business owner	4
Tailor	4
Village leader	4
Agriculture	6
*Monthly income (Tanzanian Shillings)*	
Less than 100,000	22
100,000–300,000	101
300,000–500,000	10
Above 500,000	15
*Owns a mobile phone*	
Yes	148
No	0
*Owns a smartphone*	
Yes	61
No	87
Total	148

Thematic analysis of the focus group discussions conducted with 81 participants identified two main themes (a) reasons for wanting to take part in the trial; (b) support required for trial to be a success and there were five sub-themes. Table 2 presents an overview of the coding structure.

**Table 2:** Description of the coding tree

Main themes	Sub themes
Reasons for wanting to take part in the trial	To be a citizen scientist To receive feedback on study’s outcomes To receive a mosquito bed net To receive a monetary incentive (money or airtime credit)
Support required for trial to be a success	Money for electricity to charge HumBug sensor, request for solar chargers and additional mosquito bed nets

Our findings from the qualitative analysis and illustrative quotes are presented below with relevant demographic data for participants’ gender (female = F, male = M), profession and village number. The findings are reported in line with the COREQ checklist.

### Reasons for wanting to take part in the trial

#### To be a citizen scientist

Almost half of the participants that attended the first set of focus group discussions (37 out of 81), explained that their main motivation to take part in the study was to gain enough knowledge about the types of mosquitoes that cause harm so that, firstly, they can act to protect themselves, and secondly, with the knowledge gained, they can work towards eradicating the dangerous types of mosquitoes. Responses included, for example, the following:

*“…I am interested in findings ways to identify the mosquitoes that are causing us problems in our village. Eventually, I would like to eradicate these dangerous mosquitoes. Doing this, will help us save money, compared to treating malaria…”* (F, Farmer, village 4).

*“…Taking part in this study will enable me to learn which mosquito species exist in our village and once we are able to identify them, learn how to eradicate these species…”* (M, Farmer, village 4).

#### To receive feedback on study’s outcomes

About half of the participants (38 out of 81), emphasised the importance of receiving feedback after the trial came to an end. These included: wanting to be recognised or appreciated for participating in the trial and to know whether their contribution would make a positive impact on the study; wanting to receive feedback on whether they had done a good job and wanting feedback so that they could continue working for their communities. However, the most common response (12 out of 38) referred to wanting to receive information on the study’s outcomes so the participant could protect themselves, their families, and their communities against the dangerous species of mosquitoes. Examples of each of these responses are illustrated by the following:

*“…I would like to be informed on the study’s results, because this is a new device and, I would like to know how my participation has helped with the study…”* (M, Farmer, village 1).

*“…I would like to receive feedback from you with regards to how well (or not) we have carried out the tasks for the study…”* (F, Farmer, village 1).

*“…We want everyone who has participated in this study to be provided with answers so that we can take precautions against these mosquitoes…”* (F, Farmer, village 4).

#### To receive a mosquito bed net

On the other hand, a couple of participants stated that their main motivation for taking part in the trial was that they were going to receive a mosquito bed net which they were able to keep for themselves and their families. When asked for the reason of their motivation, they stated that a mosquito bed net would protect them from mosquitoes, as illustrated by the following participant:

*“…the only thing that would motivate me is to receive a mosquito net…this is because, the mosquito net would protect me from mosquitoes that transmit malaria…”* (F, Business owner, village 1).

*“…receiving a bed net would motivate me to take part in the study…”* (F, Farmer, village 1).

#### To receive a monetary incentive (money or airtime credit)

Interestingly, only three (out of 81 people) stated that their main motivation to take part in the trial would be the provision of a monetary incentive. This could either be in the form of money or airtime credit, as illustrated by the three participants below:

*“…Yes, we need to be provided with a small amount of money, so that we can purchase some food…”* (F, Business owner, village 2).

*“…I would be interested in being in the trial group that receives airtime package because currently my life has become very difficult…”* (M, Business owner, village 1).

*“…An allowance is needed so that it would motivate me to get out of bed...!”* (F, Farmer, village 2).

In contrast, six participants stated that they would participate in the trial even if they were not offered any form of monetary incentives. This is because they felt that taking part in the study would go beyond themselves as individuals and instead, benefit the wider community, as beautifully illustrated by the following participants:

*“…I will participate in the study, even if I don’t receive an incentive. Maybe I should add one thing. An incentive is a gift. So, even if you don’t receive a gift, you can work because at the end of the day we look at what the purpose of the study is? Often research is done to help the community, so we will do it because it will benefit all community members…”* (F, Farmer, village 3).

“*…You know, in this study we are not looking at the interest of individuals, but we are looking at the interest of Tanzanians, in general…my happiness will be to represent my fellow Tanzanians…”* (M, Farmer, village 3).

### Support required for trial to be a success

#### Money for electricity to charge HumBug sensor, request for solar chargers and additional mosquito bed nets

When asked what support the participants would require for the trial to be successful, two people stated that they required a small amount of money to charge the HumBug sensors, as they did not want to go out of pocket every time, they needed to charge their sensors. In contrast, seven participants wanted to know whether it was possible to provide their households with a solar panel before the start of the trial so that they could charge their sensors, especially during power outages that frequently take place in their villages, as illustrated by a couple of participants:

*“…I have power cuts in my house, so I will need a power bank or solar panel for charging the phone…”* (M, Farmer, village 3).

*“…We often have power cuts. Sometimes for three days in a row. You can help us with a power bank, which will be a very good thing…”* (M, Business owner, village 1).

A couple of participants stated that they required an additional mosquito bed net as a form of support to help them get through the rainy season:

“…I would suggest that we have at least two bed nets, because during the rainy season it is difficult to dry our clothes when we wash them, including our mosquito net…” (F, Business owner, village 3).

“…I would like two mosquito nets because sometimes when it rains, it will be difficult to use one mosquito net…” (F, Farmer, village 4).

### Stage two: quantitative empirical study

As indicated by Fig. 2, during the first few weeks of the trial, most participants across the four trial groups switched on their sensors and uploaded data at least once on the requested dates over the 14-week trial period. For example, in week one, 92% of the participants belonging to the ‘SMS reminders and monetary incentives’ group; 89% of the participants belonging to the ‘control’ group, 81% of the participants belonging to the ‘SMS reminders only’ group and 86% of the participants belonging to the ‘monetary incentives only’ group switched on their sensors and uploaded data. However, in weeks 12, 13 and 14, perhaps, as research fatigue set in, the number of uploads across three (out of four) trial groups (‘SMS reminders and monetary incentives’, ‘control’ and ‘SMS reminders only’) started to decline.

Our analysis to ascertain the statistical significance of activity between participants belonging to the control group, compared to those in the three intervention groups (Fig. 3) indicated significant differences in switching their HumBug sensors on or off and uploading the data, between the intervention group and the control group. In particular, we found that participation (i.e., recording and uploading mosquito sound data via their HumBug sensors) was significantly worse for all incentive groups on average, compared to the control group. Interestingly, only in the case of ‘monetary incentives only’ trial group was an improved participation apparent, compared to the control group, in the last two weeks of the trial period.

### Stage three: quantitative feedback survey

At the end of the quantitative empirical study, feedback surveys were conducted to explore participants’ perspectives on their participation in the study and to capture their experiences of using the HumBug sensor.

A summary of participants’ responses is provided in Table 3.

**Table 3:** Type of response by village participants

Village name	Trial group	Strongly agree	Agree	Not sure	Disagree	Strongly disagree	Total
		n (%)	n (%)	n (%)	n (%)	n (%)	n (%)
*Statement 1: I feel that the amount of monetary incentive provided was enough (N* = *74)*							
Kivukoni	SMS reminders and monetary incentives	25 (33.8)	1 (1.4)	0	6 (8.1)	5 (6.8)	37 (50)
Milola	Monetary incentives only	8 (10.8)	14 (18.9)	0	13 (17.6)	2 (2.7)	37 (50)
	Total	33 (44.6)	15 (20.3)	0	19 (25.7)	7 (9.5)	**74 (100)**
*Statement 2: I feel that after recording, sending data using the Mozzwear application was easy (N* = *148)*							
Kivukoni	SMS reminders and monetary incentives	18 (12.2)	4 (2.7)	1 (0.7)	12 (8.1)	2 (1.4)	37 (25)
Minepa	Control	21 (14.2)	11 (7.4)	0	3 (2.0)	2 (1.4)	37 (25)
Mavimba	SMS reminders only	13 (8.8)	1 (0.7)	0	14 (9.5)	9 (6.1)	37 (25)
Milola	Monetary incentives only	12 (8.1)	13 (8.8)	0	11 (7.4)	1 (0.7)	37 (25)
	Total	64 (43.2)	29 (19.6)	1 (0.7)	40 (27.0)	14 (9.5)	**148 (100)**
*Statement 3: I would consider uploading the Mozzwear application on to my personal smartphone to collect mosquito sound data (n* = *148)*							
Kivukoni	SMS reminders and monetary incentives	36 (24.3)	1 (0.7)	0	0	0	37 (25)
Minepa	Control	16 (10.8)	6 (4.1)	12 (8.1)	3 (2.0)	0	37 (25)
Mavimba	SMS reminders only	33 (22.3)	1 (0.7)	0	2 (1.4)	1 (0.7)	37 (25)
Milola	Monetary incentives only	24 (16.2)	10 (6.8)	2 (1.4)	0	1 (0.7)	37 (25)
	Total	109 (73.6)	18 (12.2)	14 (9.5)	5 (3.4)	2 (1.4)	**148 (100)**
*Statement 4: I would like to receive feedback on the number of mosquitoes surrounding my home *via* my personal smartphone (N* = *148)*							
Kivukoni	SMS reminders and monetary incentives	33 (22.3)	4 (2.7)	0	0	0	37 (25)
Minepa	Control	30 (20.3)	6 (4.1)	0	1 (0.7)	0	37 (25)
Mavimba	SMS reminders only	36 (24.3)	0	0	1 (0.7)	0	37 (25)
Milola	Monetary incentives only	29 (19.6)	8 (5.4)	0	0	0	37 (25)
	Total	128 (86.5)	18 (12.2)	0	2 (1.4)	0	**148 (100)**
*Statement 5: I would like more information about the biology of mosquitoes (N* = *148)*							
Kivukoni	SMS reminders and monetary incentives	36 (24.3)	1 (0.7)	0	0	0	37 (25)
Minepa	Control	32 (21.6)	5 (3.4)	0	0	0	37 (25)
Mavimba	SMS reminders only	36 (24.3)	1 (0.7)	0	0	0	37 (25)
Milola	Monetary incentives	37 (25.0)	0	0	0	0	37 (25)
	Total	141 (95.3)	7 (4.7)	0	0	0	**148 (100)**

When addressing the question “was the amount of monetary incentive provided enough?” responses from 74 participants indicated 64.9% either ‘agreeing’ or ‘strongly agreeing’ that the amount was enough (Table 3: Statement 1). Questions relating to ease of use of the phone (148 participants) revealed that: the majority (62.8%) found the Mozzwear application system easy to use (Table 3: Statement 2); most of the participants (85.8%) would consider uploading the Mozzwear application on to their personal smartphones (if they had one) to collect mosquito sound data (Table 3: Statement 3); and that all the participants except one (147) either ‘agreed’ or ‘strongly agreed’ to have been treated with courtesy and respect throughout the research period and valued for taking part in the study. Almost all the participants (98.7%) either ‘agreed’ or ‘strongly agreed’ to receiving feedback on the number of mosquitoes surrounding their homes via a personal smartphone (Table 3: Statement 4), and, all the participants (148) either ‘agreed’ or ‘strongly agreed’ to wanting more information about the biology of mosquitoes (Table 3: Statement 5).

When the participants were asked what was positive about their research experience, 23.6% (35 out of 148) participants stated that gaining knowledge on the different types of harmful mosquitoes that are commonly found near their homes; 22.3% (33 out of 148) participants stated that using technology in research, recording, and sending mosquito sounds via smartphones; 19.6% (29 out of 148) participants asserted that good cooperation, relationship and trust formed between local researchers and participants; 18.2% (27 out of 148) asserted that training provided to take part in the research and learning whilst conducting the research were the positive aspects about their research experience.

Finally, when the participants were asked what would have made their research experience better, 25% (37 out of 148) participants asserted that if they were given cash (instead of a scratch card worth $10 or 23,000 Tanzanian Shillings of airtime credit), their research experience would have been better. Out of these 37 participants, nine belonged to the ‘SMS reminders and monetary incentives’ and ten belonged to the ‘monetary incentives only’ groups. A small number of participants (four out of 37) that belonged in the ‘SMS reminders and monetary incentives’ group stated that their research experience would have been better if they received an increased amount of airtime credit. Participants belonging to the ‘control’ group (one out of 37) and ‘SMS reminders only’ group (five out of 37), stated that they would have liked to have received some money for their contribution. A smaller proportion of the participants 16.2% (24 out of 148) stated they would have liked to have kept the HumBug sensors after the trial ended for personal use and 12.8% (19 out of 148) participants stated that they wanted solar chargers to help them charge their HumBug sensors.

## Discussion

The question this research set out to address was what incentives would encourage local communities to collect and upload mosquito sound data using smartphones? Our first finding was that the provision of incentives does not necessarily lead to enhanced use of the HumBug sensor. Our second finding was that the intrinsic motivational factor of gaining knowledge on the presence of harmful mosquitoes was an important driver. These findings are consistent across all three methodologies used in this study, and there were no discordant findings between the methods used. These findings will be discussed in turn.

Findings from the quantitative empirical study (i.e., stage two) revealed that the participants belonging to the three trial groups (‘SMS reminders only’, ‘Monetary incentives only’ and ‘SMS reminders and monetary incentives’) uploaded fewer data, compared to the control group. This suggests that other reasons were more important for use of the mobile app system than these three incentives. These findings contrast with previous studies that have reported that the deployment of monetary incentives and/or sending SMS reminders improved outcomes [15–23, 25]. Interestingly, only in the case of ‘monetary incentives only’ trial group did we see a significantly improved participation, compared to the control group, in the last two weeks of the trial period.

In comparison, our qualitative data analysis revealed that many participants had more than one motivational factor to take part in a citizen science project and these motivational factors, similar to Finkelstien’s (2009) findings can be placed into two groups: intrinsic and extrinsic [27].

In terms of intrinsic motivation, qualitative data analysis revealed that for many participants (30 out of 81), the main motivation for them to take part in the study in the first place was to learn more about the types of mosquitoes that cause harm so that they can act to protect themselves and their families, and work towards eradicating the dangerous types of mosquitoes. These findings are consistent with other studies on the main motivational factors of why people take part in citizen science projects [27–31]. Several studies that have been conducted in the context of high and low-income countries, reveal that the participants’ main motivation for taking part in research was the desire to learn new things [28, 29, 31, 51, 52]. Our findings are also in line with a recent qualitative study on people’s motivational factors in a citizen science programme for malaria control in rural Rwanda [31]. Here, results revealed that one of the main reasons that the participants decided to take part was because, they wanted to learn about the biology of mosquitoes, so that the mosquitoes could be easily identified by the participants. In addition, this previous study also revealed that the participants wanted to make positive contributions towards malaria reduction because they felt that they had a social responsibility to help others within their local communities from the devastating impacts of malaria. In terms of the participants requiring recognition, this study also found that some participants indicated they wanted recognition in the form of receiving feedback, rather than receiving a monetary incentive [31].

Our research also found that the participants emphasised on the importance of receiving feedback at the end of the trial. Some participants wanted to be recognised or appreciated for participating in the trial and to know whether their contribution would make a positive impact on the study. Several participants also stated wanting to receive feedback so that they would know whether they had done a good job and, continue working for their communities. Interestingly, however, the majority, wanted to be rewarded for their participation in the trial by receiving information on the study’s outcomes so that they can protect themselves, their families, and their communities against the dangerous species of mosquitoes. These findings build on the findings from several earlier studies that have been conducted in the field of citizen science from high and low-income countries. For example, a 2016 synthesis of key theories from the volunteering literature with examples from the environmental volunteering and citizen science literature [52], found that providing feedback on how the data from a citizen-science project is an important motivating factor for many participants. This study also found, that for participants to feel that their time is well spent, impact of their work should be communicated back to them [52].

In terms of extrinsic motivational factors, three participants (out of 81) in the FGD stated that their main motivation to take part in the study was, in fact the possibility of receiving a monetary incentive. This finding is consistent with the findings from a 2017 study that investigated intrinsic and extrinsic motivational factors of resource-poor farmers in participating in a digital science project conducted in India, Ethiopia and Honduras [50]. In this study, a few farmers from India only, asserted that they would like to receive some money in return for their contribution in the citizen science project. However, the main findings from this study revealed that the farmers from all three countries valued their contribution to scientific research and sharing information with one another to be the most important motivational factors, in participating in the study [50]. In our study, some participants also stated that their motivational factor for taking part in the quantitative empirical study (stage two), was to receive a free mosquito bed net from the research team. Although, the deployment of bed nets acted as a form of compensation for the participants’ time, some participants viewed them as an incentive to take part.

When asked what support the participants would require for the trial to be successful, two people stated that they required a small amount of money to charge the HumBug sensors, as they did not want to go out of pocket every time, they needed to charge their sensors. In contrast, seven participants wanted to know whether it was possible for us to provide their households with a solar panel. According to literature, if the conditions within a citizen science project are correct (i.e., adequate tools for conducting the study are provided) then there is a possibility that this would also encourage participants to collect data [53].

Interestingly, findings from the quantitative feedback survey (which was conducted after the stage two quantitative empirical study came to an end), revealed that when the participants were asked what would have made their research experience better, a quarter of the participants (37 out of 148) asserted that if they were given cash (instead of airtime credit), their research experience would have been better. A very small number of participants belonging to the ‘control’ and ‘SMS reminders only’ groups, stated that they would have liked to have received some money for their contribution. Nevertheless, all the participants from all four trial groups recorded and uploaded mosquito sound data consistently well throughout the 14-week trial period. Findings from the feedback survey also revealed that almost all the participants (147 out of 148) felt that they were treated with courtesy and respect throughout the research period and the same number of participants felt that they had been valued for taking part in the study. Most of the participants found the Mozzwear application easy to use and the majority said that they would consider uploading the Mozzwear application on to their personal smartphones (if they had one) to collect mosquito sound data. These findings are consistent with the findings from 2017 and 2018, where they reveal that if community engagement is conducted thoroughly and the community members’ contributions are valued by the local research team, then the community members place a lot of trust in the research team, resulting in higher levels of participation [53, 54]. In addition, the role of community leaders are also critical when it comes to engaging communities [55].

The ‘Expert Patient Programme’ developed by the NHS, relies on participants being better able to cope with long term health conditions by making them ‘experts’ on their own condition. This concept underpins the HumBug project; by providing people with more information about the mosquitoes that are transmitting malaria, they may be more inclined to manage their own mosquito control regimes – even if it is simply by being aware of the presence of malaria vectors to encourage bednet use. Indeed, one of the key issues often raised by those involved in deploying mosquito control, is not always the provision of the intervention, for example, long lasting insecticide treated bednets (LLINs), but the acceptance and correct use of the intervention. Moreover, factoring human behaviour into vector control programmes is becoming more common, and underpins integrated vector management (IVM). Integrated vector management promotes the ‘optimal use of resources for vector control’ [56] and community members are valuable ‘resources’ who can play a key role in local mosquito control through community participation (e.g., finding and removing larval habitats). Thus, our findings here, demonstrating the study communities’ desire to learn more about the mosquitoes that are transmitting malaria, is a very positive reaffirmation of how a ‘bottom-up’ approach could significantly impact malaria transmission.

Our study has certain strengths and limitations. The HumBug sensor has been shown to act as a state-of-the-art, user friendly and ethical mosquito survey tool implemented in real world context. On the other hand, our study holds a few limitations. First, during the course of conducting focus group discussions with the participants, potential social desirability response bias may have occurred, where respondents may have wanted to please the moderators or portray themselves as ‘good’ citizens in front of other participants, which could have modified their responses. Second, the process in which we conducted the treatment per village randomisation. We understand that by assigning a treatment per village, the variables in the different villages may be different to one another, thus influencing the outcomes. During the study design stage, we had several very long discussions about the limitations and in the end still decided to go with the current design, because we wanted to avoid conflicts between citizens of the same villages with regards to the receipt of incentives. However, post-study feedback from participants revealed that they did not care much about the incentives, and as a result, would not have harboured any resentments towards their neighbours, should they have received incentives instead of them. Third, in this study, we did not scrutinise the reasons behind the rates of mosquito sound uploads in the control group due to limited resources, as a result, we do not know why the control group had the highest upload rates. This would be an important thing to include in future work. Fourth, potential bias in the study could have been caused by the fact that once a month, the local research team in Tanzania visited the participants in all the trial groups to provide them with $2(equivalent to 4,600 Tanzanian Shillings) worth of money so that the participants could buy data which they needed to continue uploading mosquito sound data at least once a week throughout the 14-week trial period. Fifth, the participants had to use their own money and as a result had to go out of pocket to charge their Humbug sensors throughout the whole trial period. However, after the trial ended, all participants were fully reimbursed. Sixth, participants found themselves forgetting to switch off their HumBug sensors after they uploaded the mosquito sound data, resulting in battery drainage and data wastage.

## Conclusions

This study builds on an emerging body of research to indicate that intrinsic motivational factors are often more important for gaining participation in mobile phone citizen science projects than financial rewards and/or SMS reminders. From our study it would appear that intrinsic motivational factors such as that a desire to learn more about the species of mosquitoes in their communities and houses because of their role as vectors of malaria, was an important motivation. Individuals stated that this information was required for them to take protective actions for themselves and their families. These findings raise several important new research avenues, including how best to communicate back to the individuals involved, real-time information on the abundance and types of mosquitoes identified and the risk they pose. Consideration and research are needed to determine what format and style is most accessible given the many different languages, cultures, and levels of literacy in the regions of the world where mosquitoes are vectors of some of the deadliest diseases.

## Supplementary Information

**Additional file 1**: Topic guide for community members. HumBug: Developing a mosquito monitoring tool for Least Developed Countries – Focus group/interview guide to engage community members in rural Tanzania

**Additional file 2**: Topic guide for community members. HumBug: Developing a mosquito monitoring tool for Least Developed Countries – Focus group/interview guide to engage community members in rural Tanzania in Kiswahili.

**Additional file 3**: Demographic questionnaire. Do incentives improve local community collection of mosquito sound data using smartphones. Two case studies in Tanzania and the Democratic Republic of Congo. Demographic Questionnaire.

**Additional file 4**: Demographic questionnaire. Do incentives improve local community collection of mosquito sound data using smartphones. Two case studies in Tanzania and the Democratic Republic of Congo. Demographic Questionnaire in Kiswahili.

**Additional file 5**: Research Participant Experience Survey. Do incentives improve local community collection of mosquito sound data using smartphones? Two case studies in Tanzania and the Democratic Republic of Congo. Research Participant Experience Survey.

**Additional file 6**: Research Participant Experience Survey. Do incentives improve local community collection of mosquito sound data using smartphones? Two case studies in Tanzania and the Democratic Republic of Congo. Research Participant Experience Survey in Kiswahili.

## Data Availability

To protect participant anonymity, datasets are not publicly available. Anonymised datasets analysed in this study, however, are available from Professor Kathy J. Willis upon reasonable request.

## Abbreviations

COREQ: Consolidated criteria for reporting qualitative research
DHS: Demographic and Health Survey
FGDs: Focus group discussions
GSMA: Groupe Spéciale Mobile Association
IVM: Integrated vector management
LLINs: Long-lasting insecticidal nets
LMICs: Low and middle-income countries
NHS: National Health Service
NMCPs: National Malaria Control Programmes
ODK: Open Data Kit
SMS: Short Message Service
SSA: Sub-Saharan Africa
