# Supplementary material for: What incentives encourage local communities to collect and upload mosquito sound data by using smartphones? A mixed methods study in Tanzania
Source: Glob Health Res Policy. 2023 May 29;8:18. doi: 10.1186/s41256-023-00298-y (PMC10226264; doi:10.1186/s41256-023-00298-y)
Supplement: Supplementary file 1 — Additional file 1: Topic guide for community members. HumBug: Developing a mosquito monitoring tool for Least Developed Countries – Focus group/interview guide to engage community members in rural Tanzania [file 41256_2023_298_MOESM1_ESM.pdf]

HumBug: Developing a mosquito monitoring tool for Least Developed Countries –

Focus group/interview guide to engage community members in rural Tanzania

Entire Focus group discussion session/interview **Duration 80 mins**

Including, completing consent forms **10 mins (individually completed)**

Ensure all consent is correctly completed and begin recording

Including, completing questionnaire on demographics **15 mins (individually completed)**

[Begin the group by introducing self, purpose of the group and history of the research]
10 mins

e.g. Hi I'm ___ and I am a researcher at the University of Oxford, we also have xxx in the room who are going to help me with this discussion. We've asked you here today because we are interested in your opinions about a novel mosquito survey tool (HumBug) that we have developed. We are developing an acoustic sensor that identifies different species of mosquitos by their sound as they fly. It is available as an app used on smartphones. With your permission, we plan to test our sensors in your village, and invite you to help us with our work. We need to place our acoustic sensors on your bednets to listen out for mosquitoes that approach you during the night. We will provide the volunteers with a new bednet with a pocket where we will place the mosquito sensor (a smartphone – one per house). The study will require that you switch on the sensor prior to getting into bed/under your bednet and to check and recharge it when necessary. Otherwise its presence will have no impact on your day or night-time activity. The sensor will record all sounds (not just mosquito sounds) from 6pm-6am. In the mornings, we will require you to upload the captured sound data on to a web platform which we will provide. Automated algorithms will then detect any voice activity that is captured in the recordings and remove it at that point without any human involvement.

The sampling will be conducted over a four-month period that encompasses the peak mosquito season from 6pm to 6am.

Our long-term goal for the project is for communities to maintain and support the use of the app themselves via a network of community volunteers. We are therefore, working to understand the type of incentives that would make it attractive to become a community volunteer and any ethical considerations that may arise from this.

Consequently, we plan to conduct a randomised controlled trial (RCT) where one member of household who is willing to participate in the study will be randomised to one of the various study arms in each of the three trial groups and the control group. The four groups are the following: (a) monetary incentive, in the form of airtime credit, will be transferred to

participants' personal mobile phones each time they upload mosquito sound data on the days that they are scheduled to do so; (b) text message reminders will be sent to participants' personal mobile phones on the days that they are scheduled to collect mosquito sound data. The text message will instruct the participants to activate and to place their sensors in the pocket of their bed nets before going to bed on those nights; (c) monetary incentive and text message reminders will be sent to participants' personal phones; (d) no interventions. The RCT will run for four months.

[Establish ground rules]

There are a few things I'd like to run through before we start. Firstly, there are no right or wrong answers, but rather differing points of view. We'd like you to feel free to share your point of view even it differs from what others have said. Sometimes somebody in the group will have a strong opinion about something and you may have the exact opposite opinion. We would like to hear whatever you have to say.

Keep in mind that we are just as interested in negative comments as positive comments, and sometimes the negative comments are the most helpful.

As we're recording this discussion, please speak up and try not to speak over one another.

We'll only be using first-names here, and in our later reports there will not be any names attached to the comments.

My role here is to ask questions, listen and help facilitate the discussion. Feel free to talk with one another. There is a tendency in these discussions for some people to talk a lot and some people not to say much. But it is important for us to hear from each of you today because you have different experiences. So, I may ask for an opinion from someone if they are not saying much and move the discussion onto someone else if someone is sharing a lot, so we can make sure we hear from everyone.

We only have a limited time today and quite a lot to get through, so if someone is sharing something that is interesting, but not directly relevant to the questions we're discussing I might suggest we park the idea, I'll make a note, and if there is time at the end we can come back to it.

We'll finish around xxx.
[Ice breaker, we usually ask people to go round the room introduce themselves by their first name and say what they like to do on the weekends, start with yourself and any staff members, then go round the group members]

Let's begin.

Before the RCT begins:

FOCUS GROUP SCHEDULE/INTERVIEW GUIDE **(40 mins)**

1. Do you understand the purpose of this study?

2. Do you understand how we are going to set up the smartphone in your bednet?

3. Do you understand how you may be allocated to a trial group, if you were to take part in the study?

4. Can you please tell us who is your mobile network provider? E.g. Halotel, Airtel, Vodacom, Tigo, etc.

5. What would motivate you to take part in the study and why?

6. What help/support do you think you would need to use the smartphone in your home, as part of the trial?

7. Can you identify any potential challenges in conducting the trial in your home? What are the solutions, in your opinion?

8. What impact would the trial have on your day-to-day lives?

9. Would you like us to inform you with the progress of the RCT and at the end of the trial, with an overview of the results?
