# Supplementary material for: What incentives encourage local communities to collect and upload mosquito sound data by using smartphones? A mixed methods study in Tanzania
Source: Glob Health Res Policy. 2023 May 29;8:18. doi: 10.1186/s41256-023-00298-y (PMC10226264; doi:10.1186/s41256-023-00298-y)
Supplement: Supplementary file 2 — Additional file 2: Topic guide for community members. HumBug: Developing a mosquito monitoring tool for Least Developed Countries – Focus group/interview guide to engage community members in rural Tanzania in Kiswahili. [file 41256_2023_298_MOESM2_ESM.pdf]

HumBug: Kutengeneza zana ya uchunguzi wa mbu katika Nchi zinazo endelea.

Majadiliano yatafanyika kwa muda wa dalika 80
Kujaza fomu ya ridhaa dakika kumi
Anza kwa kujitambulisha , eleza dhumuni la utafitii huu.

Mimi ……………………………………………mtafiti kutoka Taasisi ya Afya ya Ifakara nimeambatana na mwenzangu ……... Tumewaalika leo kuweza kujadiliana na kupata maoni yenu juu ya kifaa cha kutambua aina za mbuu (HumBug) ambacho tumekitengeneza. Tumetengeneza kifaa ambacho kinaweza kutambua aina na makabila ya mmbu kwa kutumia sauti za mbuu wanaporuka karibu na kifaa hicho. Kifaa hicho kinatambulika kama app ambayo utaipata kwenye simu za smart phone. Kwa ruhusa yenu tunategemea kufanyia majaribio kifaa sensor hii katika Kijiji chenu na tunaomba ushirikiano wenu. Tutahitaji kuweka sensor hizo kwenye neti ambazo zitakuwa na mfuko mdogo wa kuweka simu ambayo itakuea na sensor hiyo ili iweze kuhesabu idadi ya mmbu amabo waliingia ndani ya nyumba husika wakati wa usiku.
Tutawapatia vyandarua zenye mifuko midogo na simu moja kila nyumba kwa watu watakao jitolea kufanya na sisi kazi hii. Utafiti huu unakutaka kuwasha simu kabla hujaingia kitandani kulala na kuhakikisha muda wote simu ile ina chaji nakuiacha simu. Uwepo wa sensor hiyo hautaweza kuathiri shughuli zozote. Sensor hiyo ita rekodi sauti zote (sio sauti za mmbu tu) kuanzia saa kumi na mbili jioni mpaka saa kumi na mbili asubuhi, lakini wakati wa kutuma data kunakifaa ambacho kitaondoa sauti za binadamu na sauti nyingine bila msaada wa binadamu na kubakiza sauti za mmbu tu.
Wakati wa asubuhi tutakuomba uzitume data zitakazokuwa zimerekodiwa kupitia njia maalamu ambayo utapatiwa maelezo yake.
Utafiti huu utafanyika kwa kipindi cha miezi minne kwa vipindi tofauti tofauti. Utafiti huu utaanza saa kumi nambili jioni mpaka saa kumi na mbili asubuhi.
Dhumuni letu katika utafiti huu ni kwa kuwa jamii iendelee kutoa ushirikiano juu ya utafiti huu wa sensor.kwa hiyo tunafanya kazi ilikujua aina ya motisha ambayo itawafanya wanajamii kuvutiwa kujitolea kufanya kazi katika utafiti huu.
Tunatarajia kufanya utafiti mkubwa ambao mtu mmoja katika familia atapewa moja kati ya a) motisha ya pesa kwa njia ya muda wa maongezi b)ujumbe mfupi ukimkumbusa mtumiaji siku na saa iliyopangwa kwaajili ya kurekodi mmbu c) pesa kwa njia ya muda wa maongezi pamoja na meseji za kumkumbusha mtumiaji d) hatuta toa motisha yoyote. Na hii itafanyika kwa miezi minne.

(Tengeneza sheria ndogo za kukiongoza kikao)

Kuna vitu vichache napenda tuvijadili kabla ya kuanza rasmi. Hakuna jibu baya wala zuri ila tofauti ya mitazamo tu. Tunahitaji utoe mawazo yako hatakama yatatofautiana na mwingine. Tutahitaji kusikia kila unacho waza juu ya utafiti huu.
Jua kuwa tunahitaji majibu yote hasi au chanya, wakati mwingine majibu hasi huwa yanaweza kuwa msaada mkubwa sana kwa anayefanya utafiti.
Jukumu langu hap ani kuuliza maswali, kusikiliza na kuongoja majadiliano. Uwe huru kuongea na jirani yako. Kunatabia katika majadiliano kama hayabaadhi ya watu kuongea sana na wengine kuto ongea la muhimu kwetu ni kumsikia kila mmoja akiongea na kutoa mawazo yake.

Tunamda mchache na tunamambo mengi yakujadiliana , tungependa tujadili yale yanayohusika na utafiti huu. Kama kunamtu atakuwa na swali njee ya haya nitaandika halafu nitalijibu baadae.

Tutamaliza saa xx
Anzisha majadiliano kwa kuwaacha wajitambulishe kila mmoja katika ukumbi. Baada yah apo

Tuanze majadiliano

Maswali ya kuongoza majadiliano

1. Je mmeelewa dhumuni la utafiti huu?
2. Je mmeelewa mnamna ambavyo tutaweka simu za kurekodia sauti za mmbu kwenye vyandarua vyenu?
3. Je mmeelewa namna ambavyo mtagawanya katika makundi kama mtashiriki katika utafiti ujao?
4. Unaweza kutuambia ni mtandao gani wa simu kati ya mitandao hii unafanya vizuri kijijini kwenu? Vodacom, halotel, airtel na tigo
5. Kitu gani kitakuvutia wewe kushiriki katika utafiti huu? Na kwanini?
6. Unafikiri ni msaada gani ungependa kuupata ilikuweza kutumia simu hii nyumbani kwako kama sehemu ya utafiti huu?
7. Je unaweza kuorodhesha changamoto ambazo tunaweza kukutana nazo wakati wa kufanya utafiti nyumbani kwako? Kwa maoni yako tunaweza kuzi tatuaje?
8. Ni athari gani zinaweza kusababishwa na utafiti huu katika majukumu yako ya kila siku?
9. Je utapenda kupewa taarifa juu ya maendeleo ya utafiti hu una mwisho wa utafiti huu ungependa kujua matokeo ya utafiti?

Uliza kama kuna mtu anaswali la kumalizia. WASHUKURU WASHIRIKI
