# Supplementary material for: What incentives encourage local communities to collect and upload mosquito sound data by using smartphones? A mixed methods study in Tanzania
Source: Glob Health Res Policy. 2023 May 29;8:18. doi: 10.1186/s41256-023-00298-y (PMC10226264; doi:10.1186/s41256-023-00298-y)
Supplement: Supplementary file 3 — Additional file 3: Demographic questionnaire. Do incentives improve local community collection of mosquito sound data using smartphones. Two case studies in Tanzania and the Democratic Republic of Congo. Demographic Questionnaire. [file 41256_2023_298_MOESM3_ESM.pdf]

**Participant Identification code: 001*

Do incentives improve local community collection of mosquito sound data using smartphones? Two case studies in Tanzania and the Democratic Republic of Congo (DRC)

Demographics Questionnaire

Hello. My name is ______________________________________. I am working with [the University of Oxford and Ifakara Health Institute]. We are conducting a survey about health and other topics in the following areas: Kivukoni, Minepa, Mavimba, and Milola in Tanzania. The information we collect will help us understand the characteristics of people that live in these areas. Your household was selected for the survey. I would like to ask you some questions about your household. The questions usually take about 15 to 20 minutes. The answers you give us will be confidential and will not be shared with anyone other than members of our survey team. You don't have to be in the survey, but we hope you will agree to answer the questions since your views are important. If I ask you any question you don't want to answer, just let me know and I will go on to the next question or you can stop the interview at any time. In case you need more information about the survey, you may contact the person listed on this card.

1. How old are you? __________years

2. What is your sex? (tick one box) ***interviewer to fill this in without asking this obvious question.***

   Male ☐

   Female ☐

3. What is your current education level? (tick one box)

   None ☐

   Early childhood education programme ☐

   Primary ☐

   Secondary ☐

   Higher ☐

   Don't know ☐

4. What is your profession? :

5. How many years have you lived in this village? (tick one box)

Since birth ☐

More than 5 years ☐

Less than 5 years ☐

6. How many years have you lived in this dwelling? (tick one box)

Since birth ☐

More than 5 years ☐

Less than 5 years ☐

7. What is your marital status? (tick one box)

Single ☐

Married ☐

Live with partner ☐

Other (give details) __________

8. How many adults are living in this household (including yourself)? (tick one box)

1-2 ☐

3-4 ☐

5-6 ☐

More than 6 ☐

Other (give details) ___________

9. How many children do you have? ___________children

10. Do your children go to school? (tick one box)

Yes ☐

No ☐

Don't know ☐

11. Who works in your household? (tick all that apply)

Me ☐

My partner or husband/wife ☐

Another family member ☐

No one ☐

Do not want to disclose ☐

12. What is the main source of drinking water for members of your household? (tick one box)

Piped into dwelling ☐

Piped to yard/plot ☐

Piped to neighbour ☐

Public tap/standpipe ☐

Tube well or borehole ☐

Protected well ☐

Unprotected well ☐

Protected spring ☐

Unprotected spring ☐

Rainwater ☐

Tanker truck ☐

Cart with small tank ☐
Surface water (river/dam/lake/pond/stream/canal/irrigation channel) ☐

Bottled water ☐

Other (specify) __________

13. What is the main source of water used by your household for other purposes such as cooking and handwashing? (tick one box)

Piped into dwelling ☐

Piped to yard/plot ☐

Piped to neighbour ☐

Public tap/standpipe ☐

Tube well or borehole ☐

Protected well ☐

Unprotected well ☐

Protected spring ☐

Unprotected spring ☐

Rainwater ☐

Tanker truck ☐

Cart with small tank
Surface water (river/dam/lake/pond/stream/canal/irrigation channel) ☐

Bottled water ☐

Other (specify) __________

14. Where is that water source located? (tick one box)

In own dwelling ☐

In own yard/plot ☐

Elsewhere ☐

Don’t know ☐

15. In the last month, has there been any time when your household did not have sufficient quantities of drinking water when needed? (tick one box)

Yes ☐

No ☐

Don't know ☐

16. Do you do anything to the water to make it safer to drink? (tick one box)

Yes ☐

No ☐

Don't know ☐

17. What do you usually do to make the water safer to drink? (tick all that apply)

Boil ☐

Add bleach/chlorine ☐

Strain through a cloth ☐

Use water filter ☐

Solar disinfection ☐

Let it stand and settle ☐

Other (specify) __________ ☐

Don't know

18. What kind of toilet facility do members of your household usually use? (tick one box)

Flush to piped sewer system ☐

Flush to septic tank ☐

Flush to pit latrine ☐

Flush to somewhere else ☐

Flush, don’t know where ☐

Ventilated improved pit latrine ☐

Pit latrine with slab ☐

Pit latrine without slab/open pit ☐

Composting toilet ☐

Bucket toilet ☐

Hanging toilet/hanging latrine ☐

No facility/bush/field ☐

Other (specify) __________

19. Do you share this toilet facility with other households? (tick one box)

Yes ☐

No ☐

20. Including your own household, how many households use this toilet facility?

Less than 10 households ☐

10 or more households ☐

Don’t know ☐

21. Where is this toilet facility located?

In own dwelling ☐

In own yard/plot ☐

Elsewhere ☐

22. In your household, what type of cookstove is mainly used for cooking?

Electric stove ☐

Solar cooker ☐

Liquefied petroleum gas/cooking gas stove ☐

Piped natural gas stove ☐

Biogas stove ☐

Liquid fuel stove ☐

Solid fuel stove ☐

Three stone stove/open fire ☐

No food cooked in household ☐

Other (specify) __________

23. What type of fuel or energy source is used in this cookstove?

Alcohol/ethanol ☐

Gasoline/diesel ☐

Kerosene/Paraffin ☐

Coal/Lignite ☐

Charcoal ☐

Wood ☐

Straw/shrubs/grass ☐

Animal dung waste ☐

Other (specify) __________

24. Is the cooking usually done in the house, in a separate building, or outdoors?

In the house ☐

In a separate building ☐

Outdoors ☐

Other (specify) __________

25. Do you have a separate room which is used as a kitchen?

Yes ☐

No ☐

26. What does this household use to heat the home when needed?

Central heating ☐

Manufactured space heater ☐

Traditional space heater ☐

Manufactured cookstove ☐

Traditional cookstove ☐

Three stone stove/open fire ☐

No space heating in household ☐

Other (specify) __________

27. What type of fuel or energy source is used in this heater?

Electricity ☐

Piped natural gas ☐

Solar air heater ☐

Liquefied petroleum gas (LPG)/cooking gas ☐

Biogas ☐

Alcohol/ethanol ☐

Gasoline/diesel ☐

Kerosene/Paraffin ☐

Coal/Lignite ☐

Wood ☐

Animal dung waste ☐

Other (specify) __________

28. At night, what does your household mainly use to light the home?

Electricity ☐

Solar lantern ☐

Rechargeable flashlight, torch or lantern ☐

Battery powered flashlight, torch or lantern ☐

Biogas lamp ☐

Gasoline lamp ☐

Kerosene or paraffin lamp ☐

Wood ☐

Animal dung/waste ☐

No lighting in household ☐

Other (specify) __________

29. How many rooms in this household are used for sleeping? __________rooms

30. Does this household own any livestock, herds, other farm animals, or poultry?

Yes ☐ If yes, please specify number and type of animal __________

No ☐

31. How many hectares of agriculture land do members of this household own?

__________hectares

32. Does your household have any of the following items? (tick all that apply)

Electricity ☐

A radio ☐

A television ☐

A non-mobile telephone ☐

A computer ☐

A refrigerator ☐

33. Does any member of this household own one or more of the following items? (tick all that apply)

A watch ☐

A mobile phone ☐

A smartphone ☐

A bicycle ☐

A motorcycle or motor scooter ☐

An animal-drawn cart ☐

A car or truck ☐

A boat with a motor ☐

34. Does any member of this household have an account in a bank or other financial institution?

Yes ☐

No ☐

35. Does any member of this household use a **mobile phone** to make financial transactions such as sending or receiving money, paying bills, purchasing goods or services, or receiving wages?

Yes ☐

No ☐

36. Does any member of this household use a **smart phone** to make financial transactions such as sending or receiving money, paying bills, purchasing goods or services, or receiving wages?

Yes ☐

No ☐

37. Does your household have any mosquito nets?

Yes ☐ If yes, please specify # __________

No ☐

38. How many months ago did your household get the mosquito net? __________months

39. Where did you get the net?

Government health facility ☐

Private health facility ☐

Pharmacy ☐

Shop/market ☐

Other ☐

Don’t know ☐

40. Did anyone in your household sleep under the mosquito net last night?

Yes ☐

No ☐

Not sure ☐

41. What was the main reason this net was not used last night?

Too hot ☐

Don’t like net shape/colour/size ☐

Don’t like smell ☐

Unable to hang net ☐

Slept outdoors ☐

No mosquito/No malaria ☐

Extra net/saving for later ☐

Other (specify) __________

42. What is the main material of the floor of your dwelling?

Earth/sand ☐

Dung ☐

Wood planks ☐

Palm/bamboo ☐

Parquet or polished wood ☐

Vinyl or asphalt strips ☐

Ceramic tiles ☐

Cement ☐

Carpet ☐

Other (specify) __________

43. What is the main material of the roof of your dwelling?

No roof ☐

Thatch/palm leaf ☐

Sod ☐

Rustic mat ☐

Palm/bamboo ☐

Wood planks ☐

Cardboard ☐

Metal ☐

Wood ☐

Calamine/Cement fibre ☐

Ceramic tiles ☐

Cement ☐

Roofing shingles ☐

Other (specify) __________

44. What is the main material of the exterior walls of the dwelling?

No walls ☐

Cane/palm/trunks ☐

Dirt ☐

Bamboo with mud ☐

Stone with mud ☐

Uncovered adobe ☐

Plywood ☐

Cardboard ☐

Reused wood ☐

Cement ☐

Stone with lime/cement ☐

Bricks ☐

Cement blocks ☐

Covered adobe ☐

Wooden planks/shingles ☐

Other (specify) __________
