# Supplementary material for: What incentives encourage local communities to collect and upload mosquito sound data by using smartphones? A mixed methods study in Tanzania
Source: Glob Health Res Policy. 2023 May 29;8:18. doi: 10.1186/s41256-023-00298-y (PMC10226264; doi:10.1186/s41256-023-00298-y)
Supplement: Supplementary file 4 — Additional file 4: Demographic questionnaire. Do incentives improve local community collection of mosquito sound data using smartphones. Two case studies in Tanzania and the Democratic Republic of Congo. Demographic Questionnaire in Kiswahili. [file 41256_2023_298_MOESM4_ESM.pdf]

Je! Motisha inaweza kuboresha ukusanyaji wa taarifa ya sauti za mbu katika jamii kwakutumia simu ya mkononi?

Hojaji ya Demografia

Habari. Jina langu ni ______________________________________. Ninafanya kazi na [Chuo Kikuu cha Oxford na Taasisi ya Afya ya Ifakara]. Tunafanya utafiti kuhusu mada za kiafya na zingine katika maeneo yafuatayo: Kivukoni, Minepa, Mavimba, na Milola nchini Tanzania. Taarifa tunayokusanya itatusaidia kuelewa tabia za watu wanaoishi katika maeneo haya. Kaya yako ilichaguliwa kwa uchunguzi. Ningependa kukuuliza maswali kadhaa juu ya kaya yako. Maswali kawaida huchukua kama dakika 15 hadi 20. Majibu unayotupa yatakuwa ya siri na hayatashirikiwa na mtu mwingine yeyote isipokuwa washiriki wa timu yetu ya utafiti. Sio lazima uwe kwenye uchunguzi, lakini tunatumai utakubali kujibu maswali kwani maoni yako ni muhimu. Ikiwa nitakuuliza swali ambalo hautaki kujibu, niambie tu na nitaendelea na swali linalofuata au unaweza kusimamisha mahojiano wakati wowote. Ikiwa unahitaji habari zaidi juu ya uchunguzi, unaweza kuwasiliana na mtu aliyeorodheshwa kwenye kadi hii.

1. Una miaka mingapi?-------mwaka?
2. Taja jinsia yako (weka alama kwenye kisanduku kimoja)mhojiwa kujaza hii bila kuuliza swali hili dhahiri.

- Mwanamke
- Mwanaume

3. Je una kiwango gani Cha Elimu??(Weka alama kwenye kisanduku kimoja)

- Hakuna
- Programu ya elimu ya utotoni
- Msingi
- Sekondari
- Juu zaidi
- Sijui

4. Taaluma yako ni nini?
5. Umeishi miaka mingapi katika kijiji hiki? (weka alama kwenye sanduku moja)

- Tangu kuzaliwa
- Zaidi ya miaka 5
- Chini ya miaka 5

6. Umeishi miaka mingapi katika makao haya? (weka alama kwenye sanduku moja)

- Tangu kuzaliwa
- Zaidi ya miaka 5
- Chini ya miaka 5

7. Je! Hali yako ya ndoa ikoje? (weka alama kwenye sanduku moja)

- Mseja
- nimeolewa/nimeoa
- Ishi na mwenzi
- Nyingine (toa maelezo) __________

8. Je! Ni watu wangapi wazima wanaoishi katika kaya hii (pamoja na wewe mwenyewe)? (weka alama kwenye sanduku moja)

- 1-2
- 3-4
- 5-6
- Zaidi ya 6
- Nyingine (toa maelezo) ___________

9. Una watoto wangapi? watoto ___________
10. Je! Watoto wako wanakwenda shule? (weka alama kwenye sanduku moja)

- Ndio
- Hapana
- Sijui

11. Nani anafanya kazi katika kaya yako? (weka alama zote zinazotumika)

- Mimi
- Mwenzangu au mme / mke
- Mwanafamilia mwingine
- Hakuna mtu
- Hawataki kufichua

12. Je! Ni nini chanzo kikuu cha maji ya kunywa kwa washiriki wa kaya yako? (weka alama kwenye sanduku moja)
13. Je! Ni nini chanzo kikuu cha maji ya kunywa kwa washiriki wa kaya yako? (weka alama kwenye sanduku moja)

- Mabomba kwa yadi / njama
- Bomba kwa jirani
- Bomba / bomba la umma

- Bomba au kisima
- Kisima kinachotunzwa
- Kisima kisichotunzwa
- Chemchemi iliyohifadhiwa
- Chemchemi isiyo salama
- Maji ya mvua
- Lori la mizinga
- Ndoo na tank ndogo

  Maji ya juu (mto / bwawa /
  ziwa / bwawa / mto / mfereji /
  kituo cha umwagiliaji)
- Maji ya chupa
- Chanzo kingine(kitaje)

14. Je! Ni chanzo gani kikuu cha maji kinachotumiwa na kaya yako kwa madhumuni mengine kama kupika na kunawa mikono? (weka alama kwenye sanduku moja)

- Mabomba ndani ya makao
- Mabomba kwa yadi / njama
- Bomba kwa jirani
- Bomba / bomba la umma
- Bomba au kisima
- Kisima kinachotunzwa
- Kisima kisichotunzwa
- Chemchemi iliyohifadhiwa
- Chemchemi isiyo salama
- Maji ya mvua
- Lori la mizinga
- tank ndogo

  Maji ya juu (mto / bwawa /
  ziwa / bwawa / mto / mfereji /
  kituo cha umwagiliaji)
- Maji ya chupa
- Chanzo kingine(kitaje)

15. Chanzo hicho cha maji kinapatikana wapi? (weka alama kwenye sanduku moja)

- Katika makao yako mwenyewe

- Katika uwanja / shamba
- Mahali pengine
- Sijui

16. Katika mwezi uliopita, je! Kumekuwa na wakati wowote kaya yako haikuwa na maji ya kunywa ya kutosha wakati inahitajika? (weka alama kwenye sanduku moja)

- Ndio
- Hapana
- Sijui

17. Je! Unafanya chochote kwa maji ili iwe salama kunywa? (weka alama kwenye sanduku moja)

- Ndio
- Hapana
- Sijui

18. Je! Kawaida hufanya nini ili kufanya maji salama kunywa? (weka alama zote zinazotumika)

- Chemsha
- Ongeza bleach / klorini
- Chuja kupitia kitambaa
- Tumia chujio cha maji
- Disinfection ya jua
- Acha kwa muda yatulie
- Nyingine(zitaje)
- Sijui

19. Je! Ni aina gani ya choo ambacho wanafamilia wako kawaida hutumia? (weka alama kwenye sanduku moja)

- Flush kwa mfumo wa maji taka ya bomba
- Flush kwa tank septic
- Choo kwa choo cha shimo
- Flush kwenda mahali pengine
- Flush, sijui wapi
- Vyoo vya shimo vilivyoboreshwa
- Choo cha shimo na slab
- Choo cha shimo bila slab / shimo wazi
- Choo cha mbolea

- Choo cha ndoo
- Choo cha kunyongwa / choo cha kunyongwa
- Hakuna kituo / kichaka / uwanja
- Nyingine (taja) __________

20. Je! Unashiriki choo hiki na kaya zingine? (weka alama kwenye sanduku moja)

- Ndio
- Hapana

21. Ikijumuisha kaya yako mwenyewe, ni kaya ngapi hutumia choo hiki?

- Chini ya kaya 10
- Kaya 10 au zaidi
- Sijui

22. Huduma hii ya choo iko wapi?

- Katika makao yako mwenyewe
- Katika uwanja / shamba
- Mahali pengine

23. Katika kaya yako, ni jiko gani la kupika ambalo hutumiwa kupika?

- Jiko la umeme
- Jiko la jua
- Gesi ya mafuta ya petroli / jiko la gesi ya kupikia
- Jiko la gesi asilia la bomba
- Jiko la biogas
- **Jiko la mafuta ya kioevu**
- Jiko imara la mafuta
- Jiko tatu la jiwe / moto wazi
- Hakuna chakula kilichopikwa nyumbani
- Nyingine (taja) __________

24. Ni aina gani ya chanzo cha mafuta au nishati inayotumika katika jiko hili?

- Pombe / ethanoli
- Petroli / dizeli
- Mafuta ya taa / Parafini
- Makaa ya mawe / Lignite
- Mkaa
- Mbao
- Nyasi / vichaka / nyasi

- Taka za wanyama
- Nyingine (taja) __________

25. Je! Upikaji kawaida hufanywa ndani ya nyumba, katika jengo tofauti, au nje?

- Katika nyumba
- Katika jengo tofauti
- Nje
- Nyingine (taja) __________

26. Je! Una chumba tofauti ambacho hutumiwa kama jikoni?

- Ndio
- Hapana

27. Je! Kaya hii hutumia nini kupasha moto nyumba inapohitajika?

- Inapokanzwa kati
- Hita ya nafasi iliyotengenezwa
- Hita ya jadi ya nafasi
- Jiko la kupika
- Jiko la jadi la kupika
- Jiko tatu la jiwe / moto wazi
- Hakuna kupasha moto katika kaya

28. Ni aina gani ya chanzo cha mafuta au nishati inayotumika katika hita hii?

- Umeme
- Gesi ya asili ya bomba
- Hita ya jua
- Gesi ya mafuta ya petroli (LPG) / gesi ya kupikia
- Biogas
- Pombe / ethanoli
- Petroli / dizeli
- Mafuta ya taa / Parafini
- Makaa ya mawe / Lignite
- Mbao
- Taka za wanyama
- Nyingine (taja) __________

29. Usiku, kaya yako hutumia nini hasa kuwasha nyumba?

- Umeme
- Taa ya jua

- Tochi inayoweza kuchajiwa tena, tochi au taa
- Tochi, tochi au taa inayotumiwa na betri
- Taa ya biogas
- Taa ya petroli
- Taa ya taa au taa
- Mbao
- Mavi / taka ya wanyama
- Hakuna taa katika kaya
- Nyingine (taja) __________

30. Je! Vyumba vingapi katika kaya hii hutumiwa kulala? __________ vyumba
31. Je! Kaya hii inamiliki mifugo yoyote, mifugo, wanyama wengine wa shamba, au kuku?

Ndio ikiwa ndio, tafadhali taja idadi na aina ya mnyama __________

32. Je! Washiriki wa kaya hii wanamiliki hekta ngapi za ardhi ya kilimo?

hekta __________

33. Je! Kaya yako ina vitu vifuatavyo? (weka alama zote zinazotumika)

- Umeme
- Redio
- Televisheni
- Simu isiyo ya mkononi
- Kompyuta
- Jokofu

34. Je! Mwanachama yeyote wa kaya hii anamiliki moja au zaidi ya vitu vifuatavyo? (weka alama zote zinazotumika)

- Saa
- Simu
- Simu mahiri
- Baiskeli
- Pikipiki au pikipiki
- Kikapu kinachovutwa na wanyama(mkokoteni)
- Gari au lori
- Boti na motor

35. Je! Mwanachama yeyote wa kaya hii ana akaunti katika benki au taasisi nyingine ya kifedha?

- Ndio

- Hapana

36. Je! Mtu yeyote wa kaya hii hutumia simu ya mkononi kufanya miamala ya kifedha kama vile kutuma au kupokea pesa, kulipa bili, kununua bidhaa au huduma, au kupokea mshahara?

- Ndio
- Hapana

37. Je! Mtu yeyote wa kaya hii anatumia simu ya kiganjani kufanya miamala ya kifedha kama vile kutuma au kupokea pesa, kulipa bili, kununua bidhaa au huduma, au kupokea mshahara?

- Ndio
- Hapana

38. Je! Kaya yako ina chandarua chochote?

Ndio ikiwa ndio, tafadhali taja # __________

Hapana

39. Je! Kaya yako ilipata chandarua miezi mingapi iliyopita? Miezi _________
40. Umepata wapi chandarua?

- Kituo cha afya cha Serikali
- Kituo cha afya cha kibinafsi
- Duka la dawa
- Duka / soko
- Nyingine
- Sijui

41. Je! Kuna mtu yeyote katika kaya yako amelala chini ya chandarua jana usiku?

- Ndio
- Hapana
- Sijui

42. Ni sababu gani kuu chandarua haikutumiwa jana usiku?

- Joto sana
- Hukupenda muonekano wa chandarua / rangi / saizi
- Hukupenda harufu ya chandarua
- Umeshindwa kutundika chandarua
- Kulala nje
- Hakuna mbu / Hakuna malaria
- Wavu / ziada ya ziada ya baadaye

- Nyingine (taja) __________

43. Je! Ni nyenzo gani kuu ya sakafu ya makao yako?

- Ardhi / mchanga
  Kinyesi
- Mbao za kuni
- Mtende / mianzi
- Parquet au mbao iliyosuguliwa
- Vipande vya vinyl au lami**(hii imenishinda)**
- Matofali ya kauri
- Saruji
- Zulia
- Nyingine (taja) __________

44. Je! Ni nyenzo gani kuu ya paa la makao yako?

- Hakuna paa
- Jani la nyasi / mtende
- Sod
- Kitanda cha Rustic
- Mtende / mianzi
- Mbao za kuni
- Kadibodi
- Chuma
- Mbao
- Nyuzi ya Calamine / Saruji
- Matofali ya kauri
- Saruji
- Shingles za kuezekea
- Nyingine (taja) _________

45. Je! Ni nyenzo gani kuu ya kuta za nje za makao?

- Hakuna kuta
- mitende
- Uchafu
- Mianzi na matope
- Jiwe na matope
- Adobe iliyofunuliwa

- Plywood
- Kadibodi
- Mbao iliyotumiwa tena
- Saruji
- Jiwe na chokaa / saruji
- Matofali
- Vitalu vya saruji
- Adobe iliyofunikwa
- Mbao / shingles
- Nyingine (taja) __________
