# Supplementary material for: What incentives encourage local communities to collect and upload mosquito sound data by using smartphones? A mixed methods study in Tanzania
Source: Glob Health Res Policy. 2023 May 29;8:18. doi: 10.1186/s41256-023-00298-y (PMC10226264; doi:10.1186/s41256-023-00298-y)
Supplement: Supplementary file 5 — Additional file 5: Research Participant Experience Survey. Do incentives improve local community collection of mosquito sound data using smartphones? Two case studies in Tanzania and the Democratic Republic of Congo. Research Participant Experience Survey. [file 41256_2023_298_MOESM5_ESM.pdf]

Do incentives improve local community collection of mosquito sound data using smartphones? Two case studies in Tanzania and the Democratic Republic of Congo (DRC).

Research Participant Experience Survey

Hello. My name is _______. I am working with [the University of Oxford and Ifakara Health Institute]. You have been asked to complete this survey because you have previously or are currently taking part in a randomised controlled study which will help us understand whether incentives improve local community collection of mosquito sound data using smartphones. This research has been reviewed by a committee of experts, the Oxford Tropical Research Ethics Committee (OxTREC) and the Ifakara Health Institute Institutional Review Board (IRB-IHI) to protect your rights and welfare. This study is sponsored by and insured via the University of Oxford and it is funded by the Bill and Melinda Gates Foundation. The reason for conducting this survey is to make research better so that people like you who take part have the best experience possible. The questions usually take about 15 minutes. The answers you give us will be confidential and will not be shared with anyone other than members of our survey team. You don't have to be in the survey, but we hope you will agree to answer the questions since your views are important. If I ask you any question you don't want to answer, just let me know and I will go on to the next question or you can stop the interview at any time.

If you are filling this in for someone else, please answer the questions from the participant's point of view.

To proceed with the survey, please provide your informed consent below.

I certify that I am 18 years of age or over. I have read and understood the participant information above. I consent voluntarily to the use of the information I provide in the understanding that it will be aggregated for purposes of analysis so that my responses will not be identified or identifiable.

☐	Yes, I consent
☐	No, I do not consent

1. How long have you been taking part in this research study?

☐	One month
☐	Two months
☐	Three months
☐	Four months
☐	Not sure

2. Is this the first research study you have taken part in?

☐	Yes
☐	No

Please rate how strongly you disagree or agree with the following statements about your research experience by selecting the box that matches your answer best.

3. **The information that I received before taking part prepared me for the study**

☐	Strongly disagree
☐	Disagree
☐	Not sure
☐	Agree
☐	Strongly agree
☐	I don’t remember

4. **I feel I have been kept updated about the research**

☐	Strongly disagree
☐	Disagree
☐	Not sure
☐	Agree
☐	Strongly agree
☐	It’s still too early to tell

5. **I know how I will receive the results of the research**

☐	No
☐	Yes, to some extent
☐	Yes

6. **I know how to contact someone from the research team if I have any questions or concerns**

☐	Strongly disagree
☐	Disagree
☐	Not sure
☐	Agree
☐	Strongly agree

7. **The researchers have valued my taking part in the research**

☐	Strongly disagree
☐	Disagree
☐	Not sure
☐	Agree

☐	Strongly agree

8. **Research staff have always treated me with courtesy and respect**

☐	Strongly disagree
☐	Disagree
☐	Not sure
☐	Agree
☐	Strongly agree

9. **I feel that the amount of monetary incentive provided was enough**

☐	Strongly disagree
☐	Disagree
☐	Not sure
☐	Agree
☐	Strongly agree
☐	Not applicable

10. **I feel that a fully charged sensor configured with a SIM card was able to record data overnight completely**

☐	Strongly disagree
☐	Disagree
☐	Not sure
☐	Agree
☐	Strongly agree

11. **I feel that after recording, sending data using the Mozzwear application was easy**

☐	Strongly disagree
☐	Disagree
☐	Not sure
☐	Agree
☐	Strongly agree

12. **I would consider uploading the Mozzwear application on to my personal smartphone to collect mosquito sound data**

☐	Strongly disagree
☐	Disagree
☐	Not sure
☐	Agree
☐	Strongly agree

13. **I would like to receive feedback on the number of mosquitoes surrounding my home via my personal smartphone**

☐	Strongly disagree
☐	Disagree
☐	Not sure
☐	Agree
☐	Strongly agree

14. I would like more information about the biology of mosquitoes

☐	Strongly disagree
☐	Disagree
☐	Not sure
☐	Agree
☐	Strongly agree

15. I would consider taking part in the research again

☐	Strongly disagree
☐	Disagree
☐	Not sure
☐	Agree
☐	Strongly agree

16. What was positive about your research experience?

17. What would have made your research experience better?

Finally, please tell us about yourself

18. Your profession *(Please check one box only)*

☐	Village executive officer
☐	Village leader
☐	Pastoralist
☐	Farmer
☐	Businessperson/Entrepreneur
☐	Teacher
☐	Fisherman
☐	Carpenter

☐	Operator
☐	Veterinary officer
☐	Other: what?

19. Gender *(Please check one box only)*

☐	Male
☐	Female
☐	Prefer not to say

20. Age *(Please check one box only)*

☐	18-30 years
☐	31-40 years
☐	41-50 years
☐	51-60 years
☐	61+ years
☐	Prefer not to say

This concludes the survey. Thank you very much for your help.
