# Supplementary material for: What incentives encourage local communities to collect and upload mosquito sound data by using smartphones? A mixed methods study in Tanzania
Source: Glob Health Res Policy. 2023 May 29;8:18. doi: 10.1186/s41256-023-00298-y (PMC10226264; doi:10.1186/s41256-023-00298-y)
Supplement: Supplementary file 6 — Additional file 6: Research Participant Experience Survey. Do incentives improve local community collection of mosquito sound data using smartphones? Two case studies in Tanzania and the Democratic Republic of Congo. Research Participant Experience Survey in Kiswahili. [file 41256_2023_298_MOESM6_ESM.pdf]

Do incentives improve local community collection of mosquito sound data using smartphones? Two case studies in Tanzania and the Democratic Republic of Congo (DRC).

Research Participant Experience Survey

Habari. Jina langu ni ____. Ninafanya kazi na Taasisi ya Afya ya Ifakara. Tumekuomba ushiriki wako katika dodoso hili kwasababu wewe ni mshiriki katika utafiti unaoendelea wa kurekodi sauti za mmbu ambao pia uatusaidia kuelewa kama motisha kwa wanajamii inasaidia kuboresha ukusanyaji wa taarifa za sauti za mmbu kwa kutumia simu janja. Utafiti huu umehakikiwa na wataalamu wa bodi za maadili kutoka chuo kikuu cha Oxford (Oxford Tropical Research Ethics Committee (OxTREC)) na Taasisi ya Afya ya Ifakara (Ifakara Health Institute Institutional Review Board (IRB-IHI) ili kulinda haki na utu wenu. Utafiti huu umefadhiiwa na Bill and Melinda Gates Foundation kwa chuo kikuu cha Oxford. Sababu ya kufanya dodoso hili kutaka utafiti ufanyike vyema ili watu watakao shiriki kama ninyi wawe na uzoefu mzuri. Maswali yatachukua dakika 15. Majibu utakayotoa yatakuwa ya usiri mkubwa na hayatwa tolewa kwa watu ambao hawaja husika katika utafiti huu. Tunaamini utakubali kujibu maswai yetu kwasababu taarifa hii niya muhimu sana. Kama nitakuuliza swali na hutopena kujibu tafadhali nijulishe ili niweze kuendelea na swali linguine. Tunaweza kusitisha utafiti huu mua wowote.

Ninathibitisha nina Zaidi ya miaka 18. Nimesoma na kuelewa maelezo hao juu, ninakubali kwa hiari matumizi ya taarifa ambazo nitazitoa kwa kuelewa kuwa wakati wakuchakata taarifa hizo haitaweza kuuikana kama ni mimi nimetoa taarifa hizo.

☐	Ndiyo, nina ridhia
☐	Hapana, siridhii

1. **Kwa muda gani umekuwa ukishiriki katika utafiti huu?**

☐	Mwezi mmoja
☐	Miezi miwili
☐	Miezi mitatu
☐	Miezi minne
☐	Sikumbuki

2. **Je hii ni mara yako ya kwanza kushiriki katika utafiti?**

☐	Ndiyo
☐	Hapana

3. **Maelekezo niliyopata kabla ya kuanza utafiti huu yaliniandaa vyema kushiriki utafiti huu**

☐	Sikubali sana
☐	Sikubali
☐	Sina hakika
☐	Nakubali
☐	Nakubali sana
☐	Sikumbuki

4. **Nafikiri nimekuwa nikihabarishwa mara kwa mara kuhusu utafiti huu**

☐	Sikubali sana
☐	Sikubali
☐	Sina hakika
☐	Nakubali
☐	Nakubali sana
☐	Bado mapema kusema

5. **Najua ni kwa namna gani nitapata matokeo ya utafiti huu**

☐	Hapana
☐	Ndiyo kwa kiasi fulani
☐	Ndiyo

6. **Najua namna ya kuwasiliana na watafiti kama nina swali au shida**

☐	Sikubali sana
☐	Sikubali
☐	Sina hakika
☐	Nakubali
☐	Nakubali sana

7. **Watafiti wamethamini ushiriki wangu katika utafiti huu**

☐	Sikubali sana
☐	Sikubali
☐	Sina hakika
☐	Nakubali
☐	Nakubali sana

8. **Siku zote watafiti wamenihudumia kwa adabu na heshima**

☐	Sikubali sana
☐	Sikubali
☐	Sina hakika
☐	Nakubali

☐	Nakubali sana

9. Ninahisi kiasi cha vocha ya motisha nilichopewa kinatosha

☐	Sikubali sana
☐	Sikubali
☐	Sina hakika
☐	Nakubali
☐	Nakubali sana
☐	Haitumiki

10. Ninafikiri utumiaji wa simu janja ulikuwa rahisi?

☐	Sikubali sana
☐	Sikubali
☐	Sina hakika
☐	Nakubali
☐	Nakubali sana

11. Unafikiri utumiaji wa programu ya kurekodi sauti za mmbu ulikuwa rahisi?/

☐	Sikubali sana
☐	Sikubali
☐	Sina hakika
☐	Nakubali
☐	Nakubali sana

12. Nitapenda kuweka programu ya Mozzwear kweye simu yangu binafsi ili kurekodi sauti za mmbu

☐	Sikubali sana
☐	Sikubali
☐	Sina hakika
☐	Nakubali
☐	Nakubali sana

13. Nitakuwa tayari kushiriki utafiti huu tena

☐	Sikubali sana
☐	Sikubali
☐	Sina hakika
☐	Nakubali
☐	Nakubali sana

14. Nini mtazamo wako chanya juu ya uzoefu wako katika utafiti huu?

15. **Vitu gani vingekufanya uisikie vizuri katika kufanya utafiti huu?**

16. **Taaluma yako** *(Please check one box only)*

☐	Mtendaji wa kijiji
☐	Mwenyekiti wa kijiji
☐	Wafugaji
☐	Wakulima
☐	Mfanyabiashara
☐	Mwalimu
☐	Mvuvi
☐	Fundi mwashi
☐	Mhudumu
☐	Afisa mifugo
☐	Nyingine: ipi?

17. **Jinsia** *(Please check one box only)*

☐	Mwanaume
☐	Mwanamke
☐	Sitapenda kusema

18. **Umri** *(Please check one box only)*

☐	Miaka 18-30
☐	Miaka 31-40
☐	Miaka 41-50
☐	Miaka 51-60
☐	Miaka 61+
☐	Sitapenda kusema

Huu ni mwisho wa mahojiano yetu asante sana kwa msaada wako.
